# Genome Sequence of *Xanthomonas arboricola* pv. Corylina, Isolated from Turkish Filbert in Colorado

**DOI:** 10.1128/genomeA.00246-13

**Published:** 2013-05-23

**Authors:** Jorge Ibarra Caballero, Marcelo M. Zerillo, Jacob Snelling, Christina Boucher, Ned Tisserat

**Affiliations:** Department of Bioagricultural Science and Pest Management, Colorado State University, Fort Collins, Colorado, USAa; Department of Computer Science, Colorado State University, Fort Collins, Colorado, USAb

## Abstract

Previously, we reported the isolation of a bacterium producing leaf spots in Turkish filbert. Here, we present the draft genome assembly of the bacterium identified as *Xanthomonas arboricola* pv. corylina. To our knowledge, this is the first published genome of this pathovar of *X. arboricola.*

## GENOME ANNOUNCEMENT

*Xanthomonas arboricola* pv. corylina is a pathogen of *Corylus avellana* L. and it also infects other species (1). In a previous report (2), we identified *X. arboricola* pv. corylina from leaf spots of *Corylus colurna* L. Here, we sequenced the genome of *X. arboricola* pv. corylina isolate NCCB100457 with 100 cycles of paired-end reads using an Illumina HiSeq sequencer at the USC Epigenomic Center. More than 13.3 million 100-base-long reads were produced for each end. We performed two genome assemblies, one using the A5 pipeline (3) and another using SPAdes v.2.3 (4). The A5 assembly has a total length of 5,227,695 bp consisting of 48 scaffolds (281 contigs), 43 of which were >500 bp, with the longest being 840,880 bp; the N_50_ is 263,170 bp and the G+C content is 65.46%. The SPAdes assembly has a total length of 5,398,516 bp placed in 810 nodes, 182 of which were >500 bp, with the longest being 263,848 bp; the N_50_ is 77,809 bp and the G+C content is 65.35%. We annotated the assembled genomes using the RAST server (5) and detected 4,452 and 4,500 coding sequences representing 445 and 449 “subsystems” for the A5 and SPAdes assemblies, respectively. Those numbers are similar to those in *Xanthomonas axonopodis* pv. citri (5,274,174 bp and 4,489 coding sequences represented in 461 subsystems), which is the most closely related organism present in the RAST database, based on nucleotide similarity. To further check the robustness of the NCCB100457 genome assemblies, we performed a BLASTn (6) search against 12 genes of *X. arboricola* pv. corylina that are commonly used as markers and that are present in the GenBank database. The genes *acnB*, *dnaK*, *fstZ*, *fyuA*, *gapA*, *qumA*, *rpoB*, and *rpoD* were detected and have 100% identity with other isolates from the same pathovar, while *fstX*, *groEL*, and 16S rRNA genes have 99% identity and *gyrB* has 98% identity.

We confirmed the presence of all 11 *hrp2* type 3 secretion system (T3SS) genes that are ubiquitous to all pathovars of *X. arboricola*; we also detected 20 out of 21 corresponding effector protein (T3E) genes that are present in all other *X. arboricola* pv. corylina isolates, including ATCC 19313, collected from *Corylus maxima* in the United States (7); the only exception is *avrBs3*, which is absent in NCCB100457. A PCR with *avrBs3*-specific primers (7) using the genomic DNA of NCCB100457 did not result in amplification, whereas amplicons were detected in the positive controls *Xanthomonas oryzae* pv. oryzae PXO99A and *X. oryzae* pv. oryzicola BLS256. The gene *xopH*, which is present in most *X. arboricola* pv. corylina isolates but not in ATCC 19313, is also absent in our isolate. We did not detect 31 T3E genes in the NCCB100457 genome that are also absent in other *X. arboricola* pv. corylina genomes (7). However, we detected a putative *avr* similar to *hpoG1* from *Xanthomonas campestris* pv. vasculorum (NCBI accession no. ZP_06487712.1) that was not reported before in *X. arboricola* pv. corylina. Differences in the arsenal of secretion systems and effectors can account for the pathogenicity and host specificity in pathogenic bacteria (8), including in isolate NCCB100457.

### Nucleotide sequence accession numbers.

This Whole-Genome Shotgun project has been deposited at DDBJ/EMBL/GenBank under the accession no. APMC00000000. The version described in this paper is the first version, accession no. APMC01000000.
